# Radial shockwave treatment promotes human mesenchymal stem cell self-renewal and enhances cartilage healing

**DOI:** 10.1186/s13287-018-0805-5

**Published:** 2018-03-09

**Authors:** Hao Zhang, Zhong-Li Li, Fei Yang, Qiang Zhang, Xiang-Zheng Su, Ji Li, Ning Zhang, Chun-Hui Liu, Ning Mao, Heng Zhu

**Affiliations:** 1 Department of Orthopedics, Sports Medicine Center, People’s Liberation Army General Hospital, Beijing, 100853 China; 2 Department of Cell Biology, Institute of Basic Medical Sciences, Tai Ping Road 27, Beijing, China; 3 BNLMS, State Key Laboratory of Polymer Physics & Chemistry, Institute of Chemistry, Beijing, China; 4 Department of Orthopedics, People’s Liberation Army Rocket Force General Hospital, Beijing, China

**Keywords:** Radial shockwave, Mesenchymal stem cell, Cartilage repair

## Abstract

**Background:**

Shockwaves and mesenchymal stem cells (MSCs) have been widely accepted as useful tools for many orthopedic applications. However, the modulatory effects of shockwaves on MSCs remain controversial. In this study, we explored the influence of radial shockwaves on human bone marrow MSCs using a floating model in vitro and evaluated the healing effects of these cells on cartilage defects in vivo using a rabbit model.

**Methods:**

MSCs were cultured in vitro, harvested, resuspended, and treated with various doses of radial shockwaves in a floating system. Cell proliferation was evaluated by growth kinetics and Cell Counting Kit-8 (CCK-8) assay. In addition, the cell cycle and apoptotic activity were analyzed by fluorescence activated cell sorting. To explore the “stemness” of MSCs, cell colony-forming tests and multidifferentiation assays were performed. We also examined the MSC subcellular structure using transmission electron microscopy and examined the healing effects of these cells on cartilage defects by pathological analyses.

**Results:**

The results of growth kinetics and CCK-8 assays showed that radial shockwave treatment significantly promoted MSC proliferation. Enhanced cell growth was also reflected by an increase in the numbers of cells in the S phase and a decrease in the numbers of cells arrested in the G0/G1 phase in shockwave-treated MSCs. Unexpectedly, shockwaves caused a slight increase in MSC apoptosis rates. Furthermore, radial shockwaves promoted self-replicating activity of MSCs. Transmission electron microscopy revealed that MSCs were metabolically activated by shockwave treatment. In addition, radial shockwaves favored MSC osteogenic differentiation but inhibited adipogenic activity. Most importantly, MSCs pretreated by radial shockwaves exhibited an enhanced healing effect on cartilage defects in vivo. Compared with control groups, shockwave-treated MSCs combined with bio-scaffolds significantly improved histological scores of injured rabbit knees.

**Conclusions:**

In the present study, we found that radial shockwaves significantly promoted the proliferation and self-renewal of MSCs in vitro and safely accelerated the cartilage repair process in vivo, indicating favorable clinical outcomes.

**Electronic supplementary material:**

The online version of this article (10.1186/s13287-018-0805-5) contains supplementary material, which is available to authorized users.

## Background

Articular cartilage is a nonvascularized tissue that contains only a sparse population of chondrocytes [1–4]. The collagen–proteoglycan matrix surrounding the chondrocytes gives the tissue its anticompressive features and enables frictionless motion during habitual loading. However, these features also make it difficult for cartilage to regenerate after injury. Therefore, various interventions have been developed to facilitate the regeneration of cartilage tissue [5, 6]. Since chondrocytes have a limited ability to renew and limited matrix production following cell expansion, stem cells have been the optimal choice for facilitating cartilage regeneration [1, 7].

Mesenchymal stem cells (MSCs) were originally identified in bone marrow and have since been found in numerous tissues, including bone, fat, and tendon [6, 8, 9]. Under certain conditions, MSCs can differentiate into numerous tissue cell types and promote tissue repair [7]. Moreover, MSCs are capable of repopulating themselves, continuously supplying seed cells for tissue regeneration. Given the innate ability of MSCs to promote tissue repair, there is rising interest in utilizing MSCs solely or MSCs combined with bio-scaffolds for the treatment of osteochondral disorders, including osteoarthritis, cartilage defects, and rheumatoid arthritis [1, 5, 10].

Extracorporeal shockwaves (ESWs), as a type of transient pressure fluctuation, have been accepted as an effective and safe method for the treatment of several diseases, especially for a wide range of musculoskeletal disorders [11–16]. In 2006, the US Food and Drug Administration (FDA) approved ESW devices for the treatment of plantar fasciitis. Based on the increasing accumulation of shockwave applications and improved shockwave-based therapy, researchers have begun to explore the regulatory effects of shockwaves on seed cells for regenerative medicine [17, 18]. However, the biological changes of seed cells following shockwave stimulation, especially the changes of stem cell characteristics, remain controversial.

Therefore, in the present study, we explored the biological characteristics of MSCs before and after direct treatment with radial shockwaves. Based on the shockwave literature and our previous findings of shockwaves in cartilage repair, we hypothesized that radial shockwaves may have a positive effect on MSC proliferation and repopulation, and that the promoting effect would accelerate the process of osteochondral healing in vivo.

## Methods

### MSC culture

Human bone marrow-derived MSCs were harvested from the posterior iliac crest under local anesthetic with a core biopsy needle aspiration system following our previous protocols [19–21]. Briefly, mononuclear cells were isolated by gradient centrifugation at 900 × *g* for 30 min on Percoll (Amersham Biosciences, Uppsala, Sweden) at a density of 1.073 g/ml and cultured at 2 × 10^5^–5 × 10^5^ cells/cm^2^ in alpha-modified Eagle’s medium (α-MEM; Invitrogen, Carlsbad, CA, USA) supplemented with 10% fetal bovine serum (FBS; HyClone, Logan, UT, USA). Nonadherent cells were removed by changing the culture medium after the initial 72 h. The adherent cells were trypsinized (0.05% trypsin at 37 °C for 5 min) when adherent cells were approximately 80% confluent. MSCs at passages 3–6 were used for experiments unless otherwise stated.

### Animals

Experimental animals were provided by the Experimental Animals Center of the Chinese People’s Liberation Army (PLA) General Hospital. Rabbits were kept in a controlled clean environment and received professional care. All of the experimental protocols were in compliance with the Animal Welfare Act and were approved by the Animal Care and Use Committee of the Laboratory Animal Research Center at the PLA General Hospital (Reference number: 2015-X11-10).

### Shockwave-MSC preparation in a floating model

Compared with traditional adherent stem cell culture systems, recent studies reported that floating culture systems are regarded as more physiologically relevant. Thus, a floating shockwave treatment system was constructed in the present study. In brief, a total of 2.5 × 10^7^ MSCs were harvested and resuspended in 25 ml of culture medium in 100-mm cell culture dishes. The radial shockwave applicator treated the floating MSCs below the surface of the liquid level. Radial shockwaves were generated by a Swiss DolorClast Master (Electro Medical Systems SA, Switzerland). Radial shockwave treatment was conducted at the following rates: continuous pulse, 1000 impulses, and 5 Hz (total treatment time, 200 s). Four groups were treated at different pressures as follows: 0 bar served as the control, whereas 1 bar, 2 bars, and 3 bars served as experimental groups. The radial-shockwave-treated MSCs were used for further biological experiments in vitro and in vivo.

### Growth kinetics and CCK-8 assays

The growth kinetics of radial-shockwave-treated MSCs and MSCs in the control groups were determined using trypan blue exclusion cell counting. In brief, MSCs were cultured in 48-well plates at 2 × 10^4^ cells/well and harvested every 2 days for hemocytometer cell counting during a period of 17 days.

The Cell Counting Kit-8 (CCK-8; Dojindo) was also used to evaluate MSC proliferation. In accordance with the manufacturer’s protocol, all MSCs were seeded in 96-well plates at 2 × 10^3^ cells/well (five wells in each group), cultured in α-MEM supplemented with 10% FBS, and added to the CCK-8 solution in a ratio of 100 ml/1 ml before incubation at 37 °C for 1 h. Absorbance was then measured at a wavelength of 450 nm using a microplate reader. In the present study, the CCK-8 experiments were performed over a time period of 17 days.

### Cell cycle and apoptosis analysis

Radial-shockwave-treated MSCs were collected, and the cell concentration was adjusted to 1 × 10^6^ cells/ml. MSCs in every group were then centrifuged at 300G for 15 min and fixed with 70% ethanol at 4 °C for 2 h. Fixed cells were washed and incubated in PBS supplemented with 100 mg/ml propidium iodide (PI) (Sigma-Aldrich), 100 mg/ml Annexin V (Sigma-Aldrich), and 20 ng/ml RNase (Sigma-Aldrich) for 20 min. Flow cytometry (BD FACSCanto) was used to measure stem cell fluorescence at an excitation wavelength of 488 nm. FacsDiva and MODFIT software were used to analyze the results.

### Colony-forming unit fibroblast formation assay

Passage 4 MSCs were trypsinized, harvested, and prepared before the shockwave treatment was applied. The MSCs in each group were adjusted to different cell numbers (1 × 10^3^, 5 × 10^3^, and 1 × 10^4^ cells /well) following the application of shockwave treatments. The method of shockwave stimulation followed previous protocols [21]. Aliquots of cell suspensions were added to six-well culture plates and were maintained in culture for 10 days. Crystal violet was used to stain the colonies, and their vertical gross appearances were imaged by digital photography.

### MSC pluripotency differentiation assay

To investigate the effects of radial shockwaves on multilineage differentiation capacity, MSCs in all groups were induced to differentiate into osteoblasts, adipocytes, and chondrocytes according to published protocols [20, 21]. For osteogenic differentiation, MSCs were cultured in 24-well plates at a density of 5 × 10 ^3^cells/cm^2^ with high-glucose DMEM (HG-DMEM; Invitrogen) supplemented with 10% FBS, 10 mM β-glycerol phosphate, 10^−7^ M dexamethasone, and 50 μM ascorbate-2-phosphate. Alkaline phosphatase (ALP) activity in MSCs was determined after 14 days of induction using an ALP assay kit (Sigma-Aldrich) following the manufacturer’s instructions. For adipogenic differentiation, MSCs were cultivated in 24-well plates at a density of 1 × 10^4^/cm^2^ with HG-DMEM containing 10% FBS, 0.5 μM 3-isobutyl-1-methylxanthine (IBMX), 10^−7^ M dexamethasone, and 10 ng/ml insulin for 14 days. Intracellular accumulation of adipocyte lipids in MSCs was determined by in-situ Oil-Red-O staining. For chondrogenic differentiation, MSCs were seeded in 24-well plates at a density of 1 × 10^4^/cm^2^ and induced by HG-DMEM containing 1% insulin, transferrin, and selenium (ITS), 10^−7^ M dexamethasone, 1 mM sodium pyruvate, 50 μ g/ml proline, 50 μM ascorbate-2-phosphate, and 20 ng/ml human transforming growth factor beta (TGF-β3) for 3 weeks. The differentiated cells were identified by in-situ toluidine staining.

### Real-time quantitative PCR analysis

Total RNA was extracted from radial-shockwave-treated and nontreated MSCs with TRIzol reagent (Fermentas) and reverse transcribed using the mRNA Selective PCR Kit (TaKaRa). In some experiments, the MSCs were cultured in osteogenic, adipogenic, and chondrogenic induction medium for 10 days before gene analysis. Human *Nanog*, *Oct-4*, *Sox-2*, *Runx-2*, *Osterix*, *CEBP/α*, *PPARγ*, *Sox-9*, and collagen type II (*Col-II*) cDNA were amplified by real-time PCR using a SYBR PCR Master Mix Kit (Sigma-Aldrich). The primer sequences are presented in Additional file 1: Table S1.

### Transmission electron microscopy analysis

To observe the ultrastructure changes of MSC post-shockwave treatment, transmission electron microscopy analysis was performed. After the radial shockwave treatment, MSCs were fixed with 2% glutaraldehyde in PBS at 4 °C. Veronal acetate buffer (pH 7.4) was then applied, and the samples were postfixed in 1% osmium tetroxide for 1 h at 25 °C. Samples were stained with uranyl acetate (5 mg/ml) and dehydrated in acetone, embedded in Epon 812 (EMbed812; Electron Microscopy Science, Hatfield, PA, USA). A Morgagni 268D transmission electron microscope (FEI, Hillsboro, OR, USA) was used to examine the ultrathin sections. The microscope was equipped with an AMT TEM camera system (AMT, Woolpit, UK), and the data were analyzed with AnalySIS software (SIS, Soft Imaging System GmbH, Munster, Germany).

### The healing effects of radial-shockwave-treated MSCs in vivo

To assess the healing effects of radial-shockwave-treated MSCs in vivo, cells were combined with polylactic-coglycolic acid (PLGA) scaffolds and implanted into the cartilage defects of a rabbit model. To prepare MSC-PLGA constructs, shockwave-treated MSCs were first seeded onto sterilized PLGA films to allow the MSCs to adhere to the films. The MSC-PLGA constructs were cultured in MSC medium for 8 h to improve cell attachment before implantation.

The model of the rabbit knee cartilage defect was designed following our previous protocol [22]. In brief, rabbits were anesthetized by peritoneal injection of ketamine/xylazine/buprenorphine. After shaving and disinfecting, the knee joint was opened under sterile conditions by medial parapatellar incision. The lateral patella was then exposed followed by 120° joint flexion. A round cartilage defect (4.5 mm in diameter) was created in the weight-bearing area of the femoral trochlear with a sterile trephine (external diameter 4.5 mm). To make a full-thickness cartilage lesion, the subchondral bone must be completely exposed.

Twenty skeletally mature and healthy New Zealand White rabbits (male or female, 3–4 months old, 2–2.5 kg body weight) were used to prepare the model. The animals were randomly divided into four different groups as presented in Table 1. In brief, in group A, cartilage defects were created on rabbit knees and received no therapy. In group B, the same cartilage defects were created and PLGA scaffolds were implanted into the bone lesion. In group C, untreated MSCs were combined with PLGA scaffolds and were implanted into the cartilage lesion. In group D, scaffolds were combined with radial-shockwave-treated MSCs and were implanted into the cartilage lesion. In groups C and D, 1 × 10^6^ MSCs were implanted into each rabbit. In all groups, the patella was repositioned after the operation, followed by closure of the knee capsule with precise suturing. Finally, the soft tissue flap and skin were closed in sutured layers. After recovery from anesthesia, all animals were allowed unrestrained daily activity in cages. In total, 80,000 U of penicillin were injected daily into all rabbits for 3 days following surgery.

**Table 1:** Treatment groups of experimental animals

Group	Number of rabbits	Treatment
A	5	Cartilage defects without any grafts
B	5	Cartilage defects with empty scaffolds
C	5	Cartilage defects with scaffolds seeded with untreated MSCs
D	5	Cartilage defects with scaffolds seeded with shockwave treated MSCs

*MSC* mesenchymal stem cell

### Pathological analysis of repaired tissues

Rabbits were sacrificed 8 weeks after surgery by intravenous injection of a lethal dose of barbiturate. The process was in compliance with the Animal Welfare Act. The femoral condyles, including the trochlear defects, were resected and harvested. After gross examination, the samples were fixed in 10% neutral buffered formalin. All of the samples were decalcified in ethylenediaminetetraacetic acid (EDTA) for 30–45 days, and the defects were dissected at an angle perpendicular to the surface of the lesion. After decalcification, all of the samples were dehydrated by successive concentrations of alcohol (ranging from 70% to absolute). After washing in xylene, the samples were embedded in paraffin and cut with a microtome (RM2016; Leica Microsystems). Paraffin blocks were cut into 2-μm sections and stained with histochemical stain including hematoxylin and eosin (H&E), Alcian blue, and Safranin O/Fast Green. To observe the cell nucleus in the repair area, 4′,6-diamidino-2-phenylindole (DAPI) staining was performed.

Type II collagen and proliferating cell nuclear antigen (PCNA) immunohistochemical stains were used to further identify the type and histologic origin of newly developed tissues. The sample sections were deparaffinized, rehydrated, and immersed in 5% H_2_O_2_ for 15 min at room temperature. The sections were then put in a pressure cooker with an EDTA retrieval solution at 140 °C for 3 min. After washing with phosphate-buffered saline (PBS) for 5 min (repeated three times), the slides were incubated at 37 °C with goat serum, followed by incubation with anti-IgG (Abcam Biotechology, USA). Subsequently, the slides were rinsed in PBS for 5 min (repeated three times) and incubated with antibodies (Beijing, China) for 20 min at 37 °C. Before incubation with horseradish peroxidase (HRP), the slides were washed again in PBS three times. Finally, the slides were incubated in 3,3′-diaminobenzidine tetrahydrochloride (DAB) solution and were observed under the microscope.

The stained sections were scored using the Modified International Cartilage Repair Society (ICRS) II histology scoring system for assessment of cartilage repair (Table 2). For assessment of vascularization in the layer of the subchondral bone, the modified version was applied. All histologic sections were observed using a dual-view microscope. Two senior doctors examined the specimens independently in a double-blind fashion, and a double-blind analyst calculated the final scores.

**Table 2:** Modified International Cartilage Repair Society scoring system for cartilage repair

Stain	Histologic parameter^a^	Score^b^
Safranin O/Fast Green	Surface/superficial architecture	Cartilage defect
		Adjacent
	Inflammatory cell infiltrate	Cartilage defect
		Adjacent
	Repair–host integration	Cartilage defect
	Basal neocartilage–bone integration	Cartilage defect
	Surface/superficial assessment	Cartilage defect
		Adjacent
	Mid/deep zone assessment	Cartilage defect
		Adjacent
	Overall assessment	Cartilage defect
		Adjacent
	Matrix staining (metachromasia)	Cartilage defect
		Adjacent
	Calcification/ossification	Cartilage defect
		Adjacent
	Subchondral bone vascularization	Bone subchondral
		Surrounding
	Presence of bone void (with or without fibrovascular tissue and without secretory lining), yes/no	Bone subchondral
Immunohistochemistry	C2: type II collagen	Cartilage defect
		Adjacent

^a^Defect is defined as the area of the original cartilage defect adjacent to the surface of the cartilage with 2 mm surrounding the defect and surrounding the sides and bottom of the defect

^b^Scores assigned on a 0–100 scale, 100 being normal

### Statistical analysis

Data are presented as mean values with standard deviations. Statistical significance was analyzed using Student’s *t* test. *P* < 0.05 was considered significant.

## Results

### Radial shockwave treatment promoted MSC proliferation but caused cell apoptosis

**Fig. 1:**
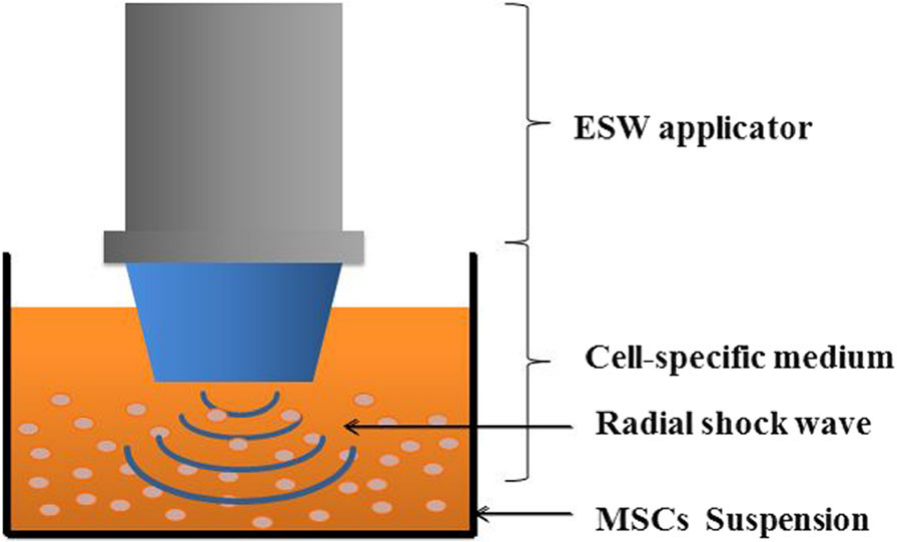
Floating system of radial shockwave stimulation. MSCs harvested and resuspended in 25 ml of culture medium in 100-mm cell culture dishes received the energy of shockwaves directly. ESW extracorporeal shockwave, MSC mesenchymal stem cell

Research has increasingly demonstrated that multipotent stem cells grown in floating culture systems exhibit enhanced angiogenic, multipotent, and tissue regenerative effects in vivo and in vitro. Therefore, in the current study MSCs were stimulated by radial shockwaves in suspension as shown in Fig. 1.

**Fig. 2:**
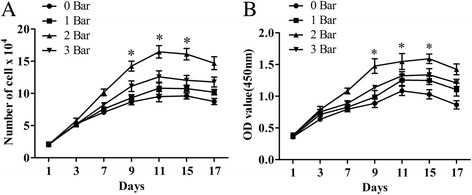
Radial shockwave stimulation promotes proliferation of MSCs in a dose-dependent manner. Trypan blue exclusion cell counting (**a**) and CCK-8-based cell proliferation assay (**b**) showed shockwave-treated MSCs exert stronger proliferative effects than control cells, and maximum cell viability occurred on the 11th day after stimulation. Optimal stimulation dose was 2 bars. Shockwave-treated MSCs incubated and measured at different pressures and compared with control groups in at least four independent experiments (different groups marked with different symbols). *Statistically significant difference compared with control group, *P* < 0.05

Cell proliferation assays and cell cycle analyses were performed to explore the effects of radial shockwaves on MSC growth. Trypan blue exclusion cell counting assays (Fig. 2a) and CCK-8 cell growth assays (Fig. 2b) demonstrated that radial-shockwave-treated MSCs exert stronger proliferative effects than MSCs in control groups. In addition, a dose–response relationship was observed with shockwave doses ranging between 0 and 2 bars, with the 2 bars group displaying the highest vitality at 11 days. The data indicated that radial shockwaves enhance MSC cell proliferation.

**Fig. 3:**
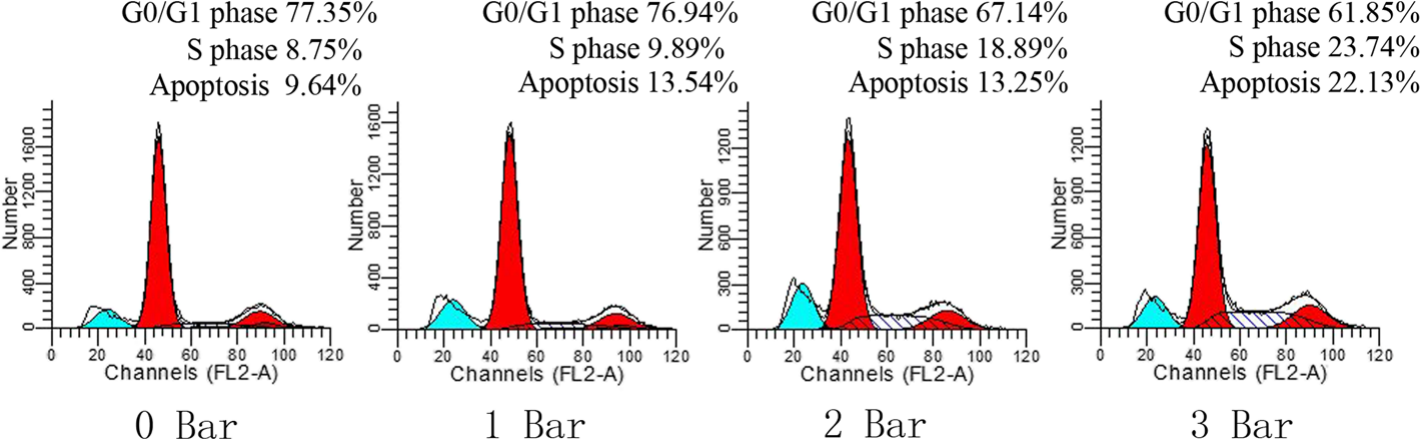
Radial shockwave influences cell cycle and apoptosis of MSC. Promotion effect reflected by cell cycle assays. Gray zones represent percentage of cells in the S phase. Higher percentage of shockwave-treated MSCs in the S phase compared to the control. On the basis of percentage of cells in the S phase and apoptosis, 2 bars is the optimal stimulation dose. Cell cycles detected by flow cytometry, and different pressures of shockwave-treated MSCs compared with control groups in at least four independent experiments. Representative data from a single experiment shown

Enhanced cell growth is also reflected in the results of the flow cytometry analyses, which showed an increased number of cells in the S phase and a decreased number of cells arrested in the G0/G1 phase (Fig. 3). Similar to the results of the trypan blue exclusion cell counting and CCK-8 assays, significant dose-dependent effects were observed for shockwaves and the percentage of cells in the S phase. There were significant differences between specific treated groups and control groups (Table 3) (0 bar vs 2 bars, *P* < 0.01; 0 bar vs 3 bars, *P* < 0.01). Thus, radial shockwave treatment might promote MSC proliferation by advancing the G1/S cell cycle transition. Moreover, the rate of apoptosis increased with increased doses of radial shockwaves. The MSCs in the 3 bars group showed the highest percentage of the apoptosis phenomenon (Table 3).

**Table 3:** Percentage in each phase of the cell cycle

Group	G0/G1 phase (%)	S phase (%)	G2 phase (%)	Apoptosis (%)
0 bars	77.15 ± 1.1	8.45 ± 0.6	13.53 ± 0.5	9.80 ± 1.2
1 bar	76.74 ± 0.7	9.49 ± 0.9	12.58 ± 0.3	14.00 ± 1.3*
2 bars	67.04 ± 1.6**	18.78 ± 2.2**	13.46 ± 0.4	13.11 ± 1.0*
3 bars	61.79 ± 3.0**	23.81 ± 1.7**	14.34 ± 0.6	22.25 ± 1.3**

Data presented as mean ± standard deviation

*Statistically significant difference compared with control groups, *P* < 0.05

**Statistically significant difference compared with control groups, *P* < 0.01

### Radial shockwave treatment increases MSC self-renewal in a dose-dependent fashion

MSCs can be distinguished from other seed cells that are involved with skeletal regeneration primarily by their multidifferentiation capacity and consistent self-renewal. These biological properties are also referred to as stemness.

**Fig. 4:**
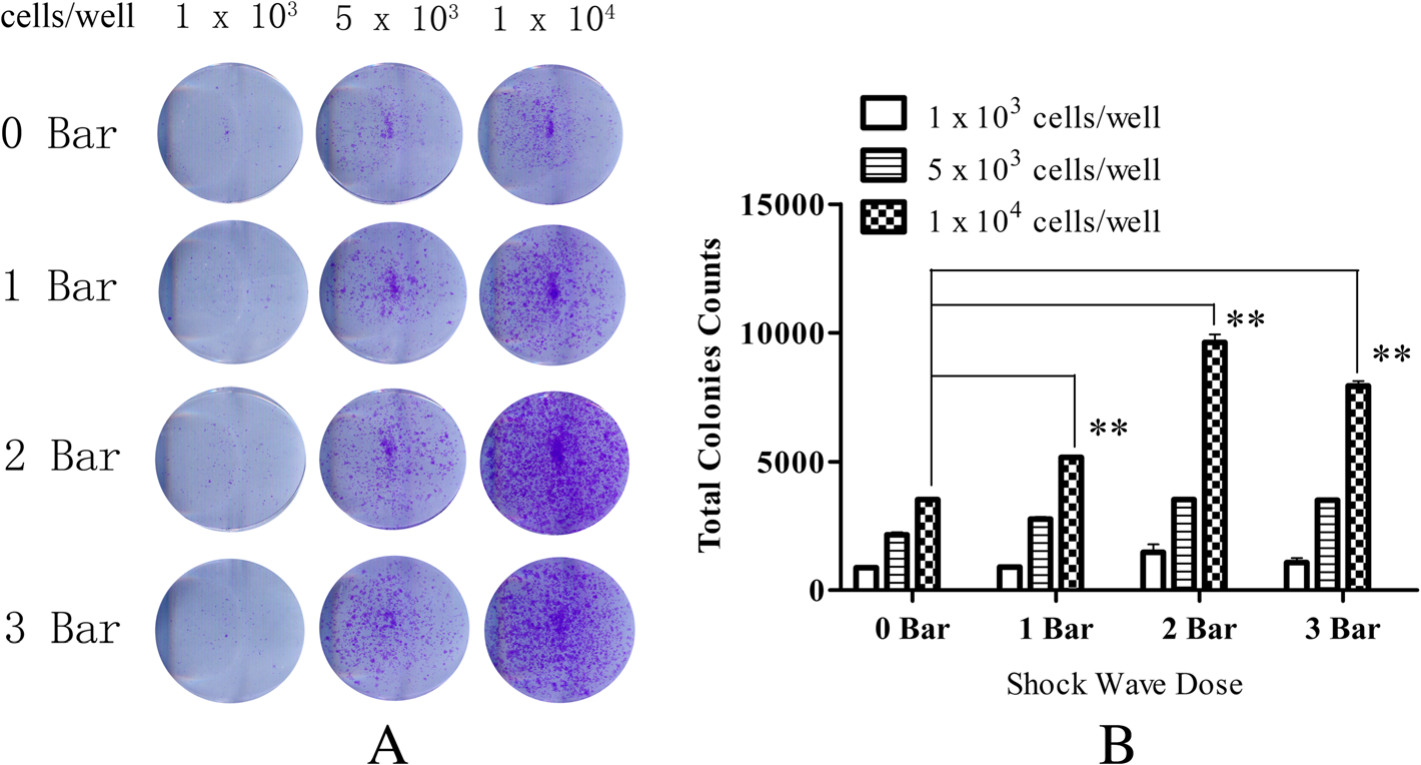
Radial shockwaves promote colony-forming unit fibroblast formation of MSCs. **a** Comparisons of colony-formation efficiency. **b** Transformed data prepared using ImageJ software. Cell number in each subgroup shown above. When cell number is 1 × 10^3^ cells, there was no significant difference among the groups. There was a slight difference when cell number is 5 × 10^3^ cells. However, there is a significant difference when cell number is 1 × 10^4^ cells; the 2 bars group showed the highest colony-formation efficiency. CFU-F formation assay performed at least three times independently; representative data from a single experiment shown. **Statistically significant difference compared with control groups, *P* < 0.001

To explore the effect of radial shockwaves on MSC self-renewal, the colony-forming unit fibroblast (CFU-F) formation test was performed. The results showed that radial-shockwave-treated MSCs generated more abundant and larger cell colonies than untreated cells (Fig. 4a, b). The promoting effects relied significantly on the shockwave dose used in the treatment (Fig. 4a, b).

**Fig. 5:**
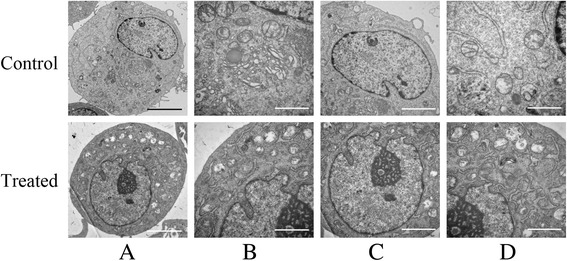
Radial shockwave treatment influence subcellular structure of MSCs. **a** Number of organelles increased. **b** In the treated group, Golgi apparatus and endoplasmic reticulum increased, and endoplasmic reticulum became increasingly well ordered and extended. **c** Nuclear diameter and area increased significantly, and kernel exhibited apparent chromosome replication. **d** Obvious increase in number of mitochondria in the treated group compared with the control. Scale bar: **a** 500 nm; **b, d** 200 nm; **c** 300 nm

To investigate the underlying mechanisms of radial shockwaves on the promotion of MSC self-renewal, MSCs and radial-shockwave-treated MSCs were analyzed with transmission electron microscopy. As shown in Fig. 5, the MSCs possessed metabolically active appearances after shockwave treatment at a dose of 2 bars. In a gross view, the cell volume is seen to have become larger, and the number of organelles is seen to have increased (Fig. 5a). In addition, the Golgi apparatus and endoplasmic reticulum were active in the treated group, and the endoplasmic reticulum became increasingly well ordered and extended (Fig. 5b). Associated with the increased nuclear diameter was a significant increase of nuclear content, and the kernel exhibited chromosome replication (Fig. 5c). In addition to the modified Golgi apparatus and endoplasmic reticulum, there was an apparent increase in the number of mitochondria in the treated group compared with the control (Fig. 5d).

In accordance with changes in CFU-F formation and subcellular structures, the results of real-time quantitative PCR showed that the transcript levels of the self-replication genes Nanog, Oct-4, and Sox-2 were markedly higher in the radial-shockwave-treated MSCs than those in the untreated MSCs (Additional file 2: Figure S1). This suggests that radial shockwave treatment enhances the self-renewing ability of MSCs.

### Radial shockwave exhibited different effects on osteogenic, adipocytic, and chondrocytic differentiation of MSCs

**Fig. 6:**
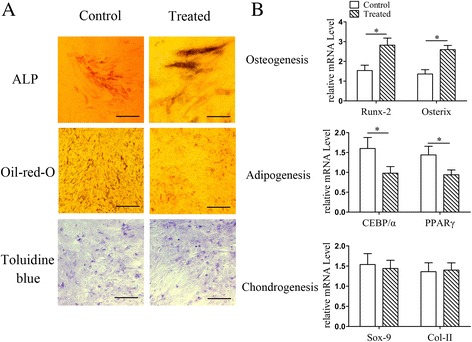
Impact of radial shockwaves on multidifferentiation of MSCs. **a** Multidifferentiation tests of MSCs showed radial shockwave stimulation increased ALP activity but decreased formation of Oil Red-O-positive lipid droplets, indicating that shockwaves promote osteogenesis induction and block adipogenesis at the same time. No significant differences in the two cohorts of cells after chondrogenic induction. **b** Impact of shockwaves on MSC differentiation also reflected by mRNA levels of *Runx-2*, *Osterix*, *CEBP/α*, *PPARγ*, *Sox-9*, and * Col-II*. Quantitative PCR assay performed at least three times independently; representative result shown. *Statistically significant difference compared with control groups, *P* < 0.05. ALP alkaline phosphatase, Col-II collagen type II

To further explore the impact of radial shockwaves on MSC stemness, multidifferentiation tests of MSCs were performed. As shown in Fig. 6, strong ALP activity was observed in radial-shockwave-treated MSCs following a 2-week period of osteogenic induction. In contrast, weaker ALP expression was observed in untreated MSCs (Fig. 6a). However, few Oil Red-O-positive lipid droplets were observed in radial-shockwave-treated MSCs, while many lipid droplets formed in MSCs of the control group (Fig. 6a). In addition, no significant differences were observed in the two cohorts of cells after chondrogenic induction (Fig. 6a).

To further determine the effects of radial shockwaves on MSC multipotency, the mRNA levels of * Runx-2*, *Osterix*, * CEBP/α*, *PPARγ*, *Sox-9*, and *Col-II* were examined by real-time PCR. Consistent with the results of the histochemical staining, the radial-shockwave-treated MSCs exhibited increased mRNA expression of *Runx-2* and *Osterix* after osteogenic induction and decreased mRNA expression of * CEBP/α* and *PPARγ* after adipogenic induction. No significant change in mRNA expression of *Sox-9* and *Col-II *was observed after chondrogenic induction (Fig. 6b). These data indicate that radial shockwaves differentially regulate MSC differentiation into osteocytes, adipocytes, and chondrocytes.

### Radial-shockwave-treated MSCs enhanced the healing effect on rabbit cartilage defects

**Fig. 7:**
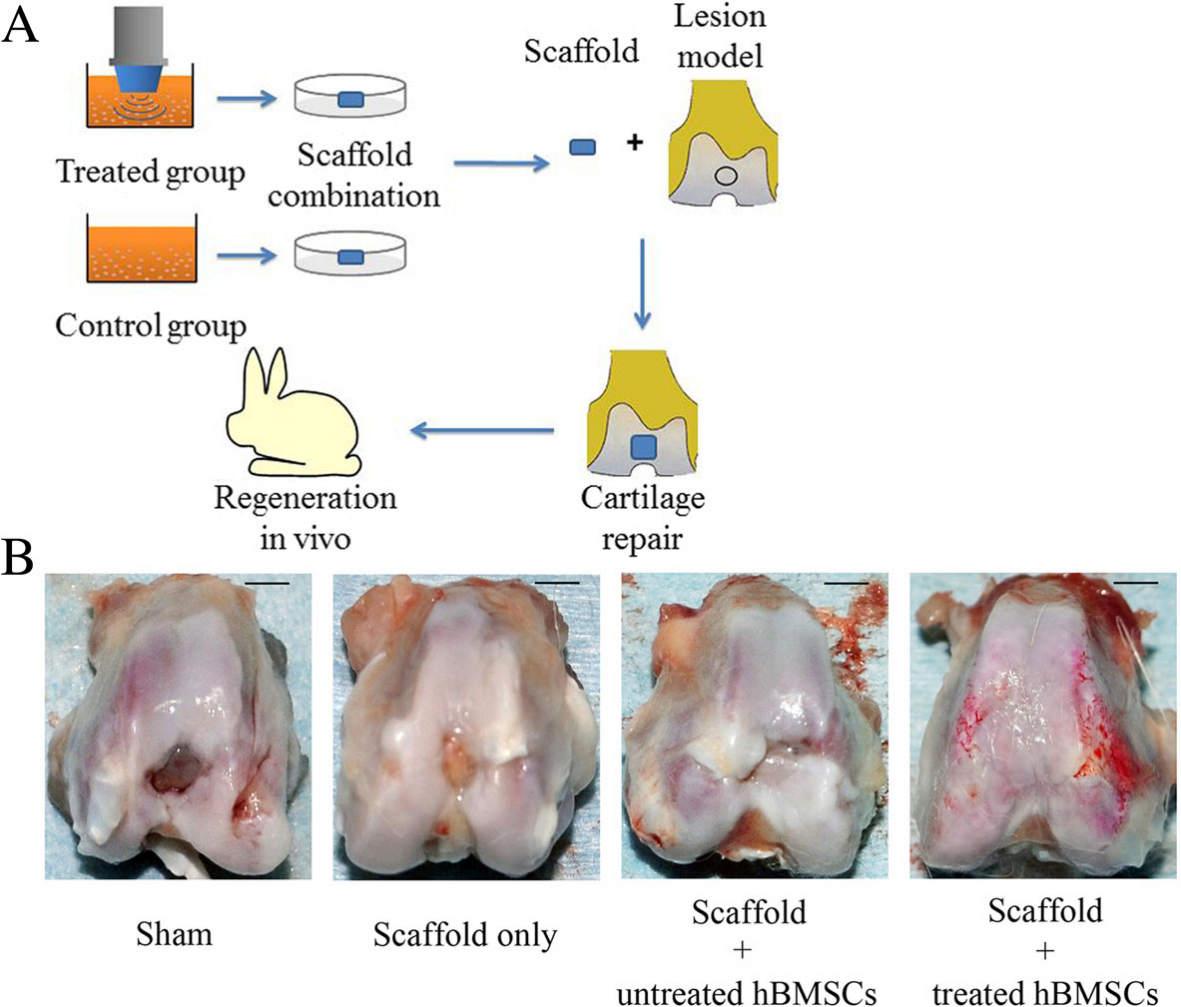
Cartilage repair model and evaluation of gross repairing effect. **a** Flow diagram of cartilage repairing experiments. **b** Gross appearance in each group. Scale bar = 5 mm. hBMSC human bone marrow mesenchymal stem cell

All animals were sacrificed at 8 weeks following surgery. As shown in Fig. 7, the cartilage repair effect of each group was clearly apparent as seen by the gross morphology. The sham surgery group showed a completely unhealed lesion and appeared to have full-thickness chondral defects. In the scaffold-only group, the base of the lesions was covered with fibrous tissues. When the scaffold was seeded with untreated MSCs, there were marked cartilage-repairing responses in the area of the lesions, but the healing processes were relatively slow and the lesions exhibited uneven articular surfaces. In the scaffold seeded with radial-shockwave-treated MSCs, the cartilage defects were completely healed. Regenerated cartilage tissue had completely filled the defect, as seen by the smooth glossy surface of the femoral trochlear (Fig. 7b).

**Fig. 8:**
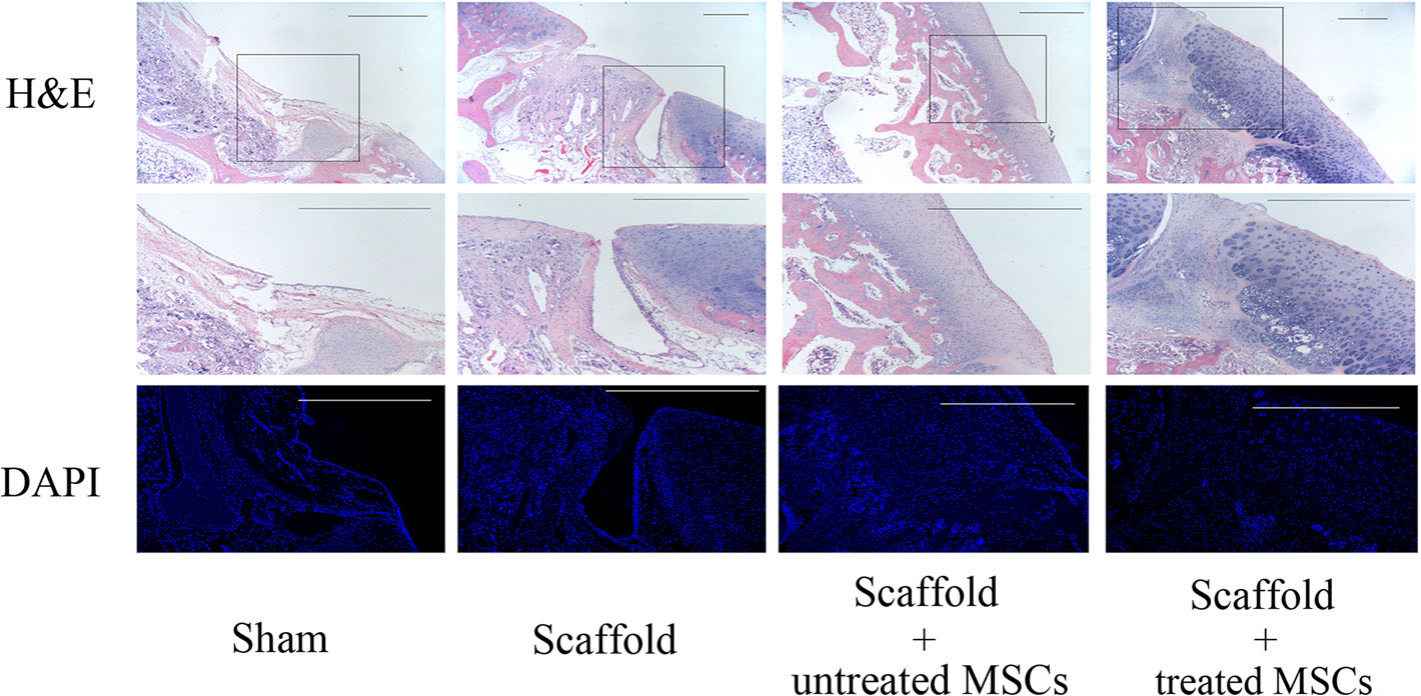
Pathological analyses of radial-shockwave-treated MSCs mediated cartilage regeneration. H&E-stained section showing integrity of repaired tissue. Indicated areas magnified to show details. Using DAPI stain, number and morphology of nucleus shown. Cartilage cells markedly increased in shockwave-treated group compared to control group. Scale bar = 1 mm. H&E hematoxylin and eosin, DAPI 4′,6-diamidino-2-phenylindole, MSC mesenchymal stem cell

**Fig. 9:**
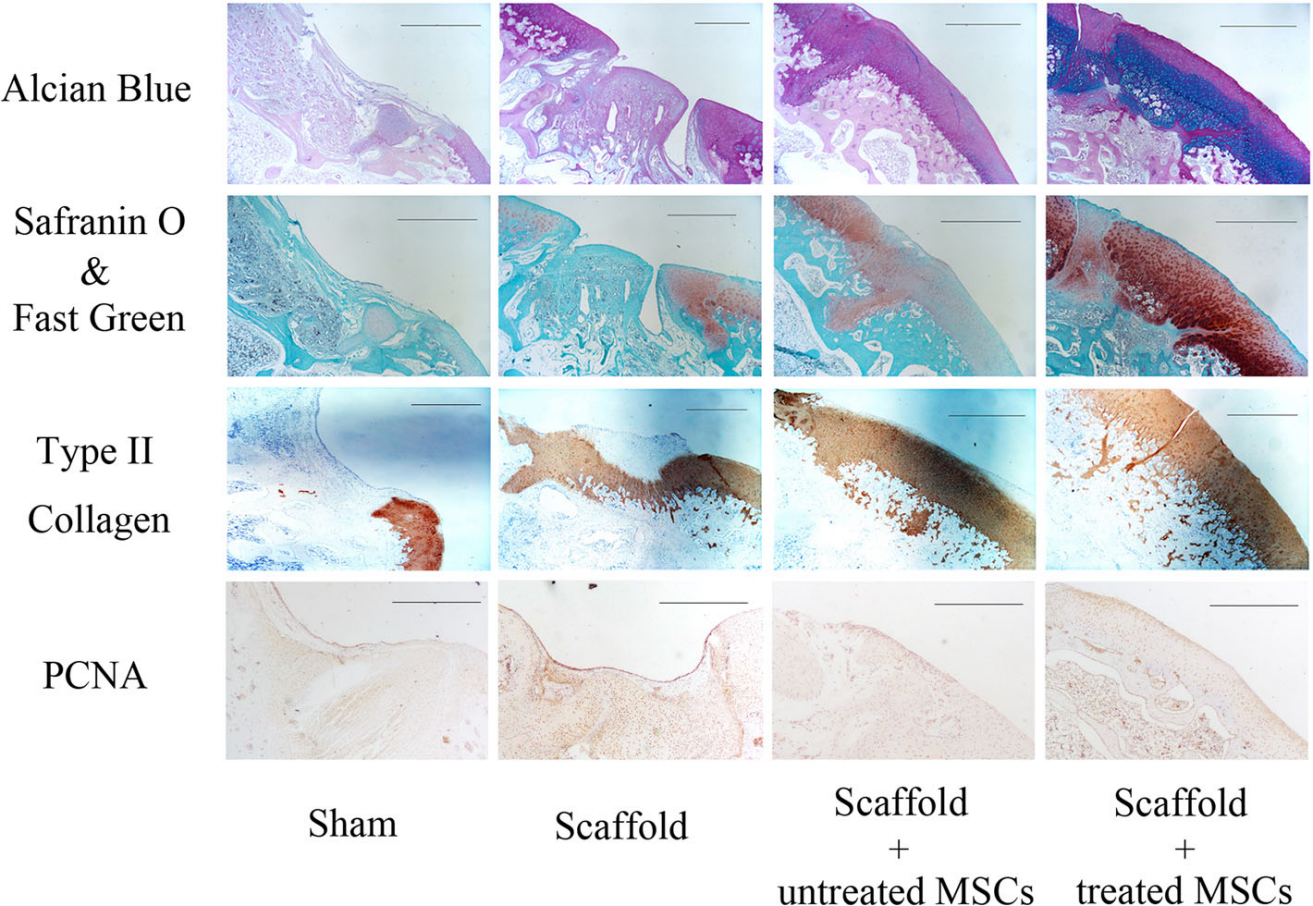
Radial shockwaves enhance formation of cartilage matrix and type II collagen in vivo. At 8 weeks following surgery, histochemical staining (Alcian blue and Safranin O/Fast Green) and immunohistochemical staining (type II collagen and PCNA) were performed. Stained sections scored using the modified ICRS II histology scoring system for cartilage repair. Scale bar = 1 mm. PCNA proliferating cell nuclear antigen, MSC mesenchymal stem cell

Further pathological analyses demonstrated the different cartilage-repairing activities of each group in detail. H&E and DAPI staining was employed to identify the general characteristics of the repaired tissues (Fig. 8). Alcian blue, Safranin O/Fast Green, and type II collagen staining showed chondrocyte differentiation, and PCNA staining revealed cell proliferation activity in vivo (Fig. 9). The histological data showed that no cartilage appeared in the defects of the sham surgery group and only a thin film of chondrocyte-like connective tissue covered the defect area implanted with the blank scaffold. In contrast, chondrocyte-like tissue filled the defects engrafted by both MSC-PLGA constructs. Most importantly, compared with the other groups, the scaffold seeded with radial-shockwave-treated MSCs showed abundant mature chondrocytes and a considerable amount of proteoglycans in newly formed cartilage tissue. The regenerative cartilage tissues in the defects of the radial-shockwave-treated group were similar to normal cartilage tissues, and the margins of the lesions were nearly absent. Moreover, the results of the PCNA staining indicated that radial shockwave treatment may result in more proliferative tissues in vivo.

In addition, we evaluated the repair of cartilage defects by the ICRS II histology scoring system for cartilage repair. Table 4 shows that new cartilage formation in the defects that were implanted with radial-shockwave-treated MSC-PLGA constructs was significantly improved compared to cartilage formation in the defects that were implanted with the untreated MSC-PLGA constructs.

**Table 4:** Significant findings on histologic section assessment

Histologic category	Sham	S only	S + untMSC	S + tMSC	*P* value
Surface/superficial architecture	23 ± 2	55 ± 3	83 ± 6	98 ± 3	0.01^**^
Repair–host integration	19 ± 7	62 ± 18	79 ± 13	87 ± 11	0.04^*^
Basal integration	25 ± 3	69 ± 5	88 ± 3	93 ± 4	0.05^*^
Overall assessment	27 ± 2	65 ± 8	83 ± 8	93 ± 6	0.03^*^
Matrix staining (metachromasia)	12 ± 4	47 ± 12	82 ± 5	98 ± 3	0.01^**^
Subchondral bone vascularization	33 ± 9	67 ± 11	83 ± 10	84 ± 8	0.2
Type II collagen	17 ± 2	71 ± 15	90 ± 5	93 ± 3	0.1

Data presented as mean ± standard deviation. The International Cartilage Repair Society II scoring system was modified to assess cartilage repair and vascularization in subchondral bone. A score of 100 indicates normal and 0 represents complete loss of normal architecture. Student’s *t* test applied to compare the S + untMSC and S + tMSC group

*Sham* sham surgery group, *S only* scaffold only group, *S + untMSC* scaffold + untreated MSC group, *S + tMSC* scaffold + treated MSC group, *MSC* mesenchymal stem cell

*Statistically significant difference compared with control groups, *P* < 0.05

**Statistically significant difference compared with control groups, *P* < 0.01

## Discussion

In the current study we demonstrated the promoting effects of radial shockwaves on cell proliferation and self-renewal. In addition, we found that radial shockwave treatment enhanced the healing effects of MSCs on cartilage defects in a rabbit model.

Although numerous reports have demonstrated that shockwaves influence MSC biological properties, the regulatory effects of shockwaves on MSCs are still disputed [18, 23–26]. The controversial data in various studies may be due to highly heterogeneous cell origins and variable shockwave application methods. To exclude the potential impact of biological media such as bio-gel and skin, a floating system was used in the current study. Suspended MSCs were stimulated by shockwaves to minimize energy loss and to reveal direct effects on MSCs. In the present study, shockwave treatment followed the previous protocol [27, 28] of continuous pulse, 1000 impulses, and 5 Hz (total treatment time 200 s).

Using this novel system, we found that radial shockwave treatment significantly enhanced MSC proliferation. Proliferation effects were confirmed by growth kinetics assay, CCK-8 assay, and cell cycle analysis. Notably, a significant dose-dependent shockwave effect was observed for doses between 0 and 2 bars; MSCs in the 2 bars group displayed the highest vitality after 11 days. In addition, the promoting effect lasted for nearly 12 h after the shockwave treatment according to previous studies. These findings suggest that it is possible to upregulate the growth efficiency of various seed cells that are associated with regenerative medicine using shockwave treatment in appropriate systems. However, our data showed that direct application of shockwaves on MSCs resulted in cell apoptosis. This result is consistent with previous reports using equine adipose tissue-derived MSCs, indicating that shockwave therapy may be a double-edged sword and inappropriate applications of shockwaves may lead to cell death.

Self-replication or self-renewal controls the stem cell pool and determines the number of skeletal progenitors. However, little information is available about the effects of shockwaves on human bone marrow MSCs. In the current study, MSC self-renewal was assessed by the CFU-F formation assay, transcription expression levels of key factors, and ultrastructural analyses of MSCs. Compared with the expression of stem cell surface markers, the CFU-F formation test assesses the colony-forming function of the cells and provides a more convincing evaluation of MSC self-renewal. Our data demonstrated that radial shockwave treatment augments colony formation of MSCs in a dose-dependent manner, strongly suggesting that these mechanistic stimuli enhance MSC self-repopulation. As expected, radial shockwave treatment resulted in increased mRNA levels of the self-renewal genes *Nanog*, *Oct-4*, and *Sox-2*, which supports the hypothesis that radial shockwaves enhance the innate self-renewing activity of MSCs [29, 30]. Although previous studies were highly concerned about changes in the biological behavior of stem cells after shockwave stimulation, little is known about the changes in the ultrastructure of these cells after treatment. In this study, we found many changes of organelle structure using transmission electron microscopy. The activation of the Golgi apparatus and endoplasmic reticulum suggests that protein synthesis increases after treatment, and the enlargement of nucleoli and mitotic figures is consistent with thriving proliferative phenomena. In addition, there was a significant increase in the number of mitochondria after shockwave stimulation, which also indicates that stem cell metabolism had increased. Taken together, the data indicate that radial shockwaves modulate the stemness of MSCs by promoting self-replicating activities.

Increasing evidence suggests that shockwaves direct stem cell differentiation; however, this hypothesis has not been fully investigated. Raabe et al. [31] reported that in-vitro shockwave treatment promotes the osteogenic, adipogenic, and chondrogenic differentiation of equine adipose tissue-derived MSCs, but our analysis of the adipogenesis-relevant, osteogenesis-relevant, and chondrogenesis-relevant mRNA expression did not show any significant differences between samples. In contrast, Suhr et al. [32] found that while the adipogenic differentiation was unaffected by shockwave treatment, the osteogenic differentiation potential was slightly reduced in vitro. In addition, they observed a more effective chondrogenic differentiation of human bone marrow MSCs after shockwave application. Moreover, Priglinger et al. [33] demonstrated that shockwave therapy significantly improved osteogenic and adipogenic differentiation of human adipose tissue-derived cells, but no difference in chondrogenic differentiation was visible between control and shockwave-treated cells. In our study, the MSC multilineage differentiation potential was evaluated after direct treatment with radial shockwaves. We found that radial shockwaves favored MSC osteogenic differentiation but inhibited the adipogenic activity of these cells. In addition, the chondrogenic capacity of MSCs was not impaired by shockwave stimuli. Our findings suggest that direct shockwave treatment is helpful for maintaining MSC differentiation capacity for bone and cartilage but might limit the regeneration of adipose tissue.

Although in-vitro data demonstrated that radial shockwave treatment significantly increased the number of MSCs and did not impair their regenerative capacity for skeletal tissues, it remains unknown whether treated MSCs were sufficiently potent to improve osteochondral formation in vivo. For improved healing in situ, porous PLGA scaffolds were used in our study to provide a surface and void volume upon which MSCs were able to attach, proliferate, and differentiate as reported previously. The general observations and histological analyses of rabbit cartilage defects indicated that radial-shockwave-treated MSCs significantly enhanced and promoted tissue repair. These results lead us to reconsider the underlying mechanisms of shockwave therapy. The data also suggest ideas for the development of novel strategies in cartilage repair by virtue of boosting MSC self-renewal. Although the mechanisms of shockwave-promoting effects on MSC proliferation and differentiation have been investigated in previous studies, the underlying mechanisms of our findings need to be revealed in further study.

There are still some limitations to the present study. Only pressure and frequency can be regulated with the radial shockwave generator that we used, thereby limiting the different dose-dependent studies to these parameters. In addition, we found that there is minor cell loss during shockwave treatment. Moreover, further investigations into the mechanisms of cell signal transduction are needed to strengthen the findings.

## Conclusion

Our studies demonstrate that radial shockwave treatment is a potential tool for regulating MSC behavior and show that these effects could be involved with MSC cartilage regeneration. Therefore, this strategy appears to have promising clinical relevance and may provide a low-cost and effective treatment for cartilage repair.

## Additional files

Additional file 1: **Table S1.** Presenting primer sequences. (DOC 34 kb)

Additional file 2: **Figure S1.** Showing radial shockwaves increase transcript levels of stemness markers. Transcript levels of self-replication genes *Nanog*, *Oct-4*, and *Sox-2* were significantly higher in the radial-shockwave-treated MSCs than in untreated MSCs (*P < 0.05). Representative data from three separate experiments shown. (TIFF 1329 kb)

## Abbreviations

ALP: Alkaline phosphatase
CCK-8: Cell Counting Kit-8
CFU-F: Colony-forming unit fibroblast
Col-II: Collagen type II
DAB: 3,3′-Diaminobenzidine tetrahydrochloride
DAPI: 4′,6-Diamidino-2-phenylindole
DMEM: Dulbecco’s modified Eagle medium
EDTA: Ethylenediaminetetraacetic acid
ESW: Extracorporeal shockwave
FACS: Fluorescence activated cell sorting
FDA: Food and Drug Administration
H&E: Hematoxylin and eosin
HRP: Horseradish peroxidase
IBMX: 3-Isobutyl-1-methylxanthine
ICRS: International Cartilage Repair Society
ITS: Insulin, transferrin, and selenium
MEM: Minimum essential medium
MSC: Mesenchymal stem cell
PBS: Phosphate-buffered saline
PCNA: Proliferating cell nuclear antigen
PI: Propidium iodide
PLA: Chinese People’s Liberation Army
PLGA: Polylactic-coglycolic acid
TGF-β3: Transforming growth factor beta
