# Supplementary material for: Radial shockwave treatment promotes human mesenchymal stem cell self-renewal and enhances cartilage healing
Source: Stem Cell Res Ther. 2018 Mar 9;9:54. doi: 10.1186/s13287-018-0805-5 (PMC5845163; doi:10.1186/s13287-018-0805-5)
Supplement: Supplementary file 1 — Table S1. Presenting primer sequences. (DOC 34 kb) [file 13287_2018_805_MOESM1_ESM.doc]

| genes | primer sequences | Annealing temperature |
| --- | --- | --- |
| HPRT  Nanog  Oct-4  Sox-2  Runx-2  Osterix  CEBP/α  PPARγ  Sox-9  Col-II | forward, 5′- CCTGGCGTCGTGATTAGTGA -3′  reverse, 5′- GCCTCCCATCTCCTTCATCA -3′  forward, 5′- TGAGTGTGGATCCAGCTTGT-3′  reverse, 5′- TCTCTGCAGAAGTGGGTTGT-3′  forward, 5′- GGTCCGAGTGTGGTTCTGTA-3′  reverse, 5′- CGAGGAGTACAGTGCAGTGA-3′  forward, 5′- CATGTCCCAGCACTACCAGA-3′  reverse, 5′- TACCGGGTTTTCTCCATGCT-3′  forward, 5′- CTGTGGTTACTGTCATGGCG-3′  reverse, 5′- CCCTCCCTTTTCCCACTCAT-3′  forward, 5′- CATGGATGCCTGCCTTGTAC-3′  reverse, 5′- CTGGTGGACCACTCGGATGA -3′  forward, 5′- GGAGGGTCTCTAGTTCCACG-3′  reverse, 5′- CCCACAGCCAGATCTCTAGG-3′  forward, 5′- TTGCAGTGGGGATGTCTCAT-3′  reverse, 5′- TTTCCTGTCAAGATCGCCCT-3′  forward, 5′- ATGAAGATGACCGACGAGCA-3′  reverse, 5′- AACTTGTCCTCCTCGCTCTC-3′  forward, 5′- AGCCTGGTGATGATGGTGAA-3′  reverse, 5′- ACTCTCACCCTTCACACCAG-3′ | 60 ℃ |

**Table S1: Primer sequences**
