# Supplementary figures and images for: Radial shockwave treatment promotes human mesenchymal stem cell self-renewal and enhances cartilage healing
Source: Stem Cell Res Ther. 2018 Mar 9;9:54. doi: 10.1186/s13287-018-0805-5 (PMC5845163; doi:10.1186/s13287-018-0805-5)

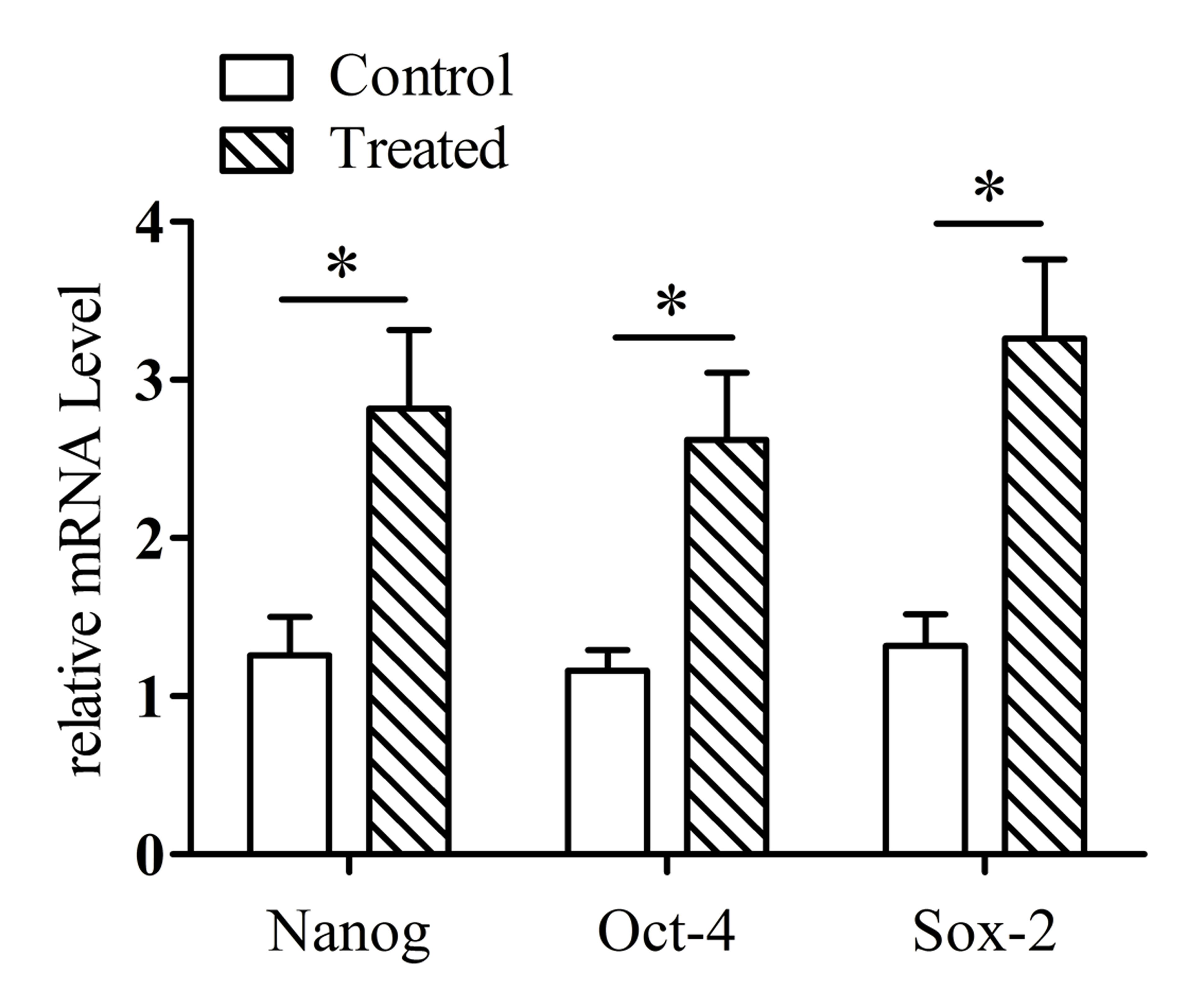

Supplement: Supplementary file 2 — Figure S1. Showing radial shockwaves increase transcript levels of stemness markers. Transcript levels of self-replication genes Nanog, Oct-4, and Sox-2 were significantly higher in the radial-shockwave-treated MSCs than in untreated MSCs (*P < 0.05). Representative data from three separate experiments shown. (TIFF 1329 kb) [file 13287_2018_805_MOESM2_ESM.tif]
